# LncRNA SNHG6 Induces Epithelial–Mesenchymal Transition of Pituitary Adenoma Via Suppressing MiR-944

**DOI:** 10.1089/cbr.2020.3587

**Published:** 2022-05-10

**Authors:** Dandan Mao, Yuanqing Jie, Yao Lv

**Affiliations:** Department of Neurosurgery, Quzhou People's Hospital, Quzhou, China.

**Keywords:** EMT, miR-944, pituitary adenoma, SNHG6

## Abstract

**Background:**

Pituitary adenoma (PA) is a common primary brain tumor with invasive properties. Despite that long noncoding RNA (lncRNA) small nucleolar RNA host gene 6 (SNHG6) exerts oncogenic function in cancer cells and that miR-944 inhibits epithelial–mesenchymal transition (EMT) of cancer cells are well documented, few studies have explored the function and mechanism of SNHG6 and miR-944 in invasive pituitary adenoma (IPA).

**Materials and Methods:**

Quantitative real-time polymerase chain reaction (qRT-PCR) was used to detect the expressions of SNHG6 and miR-944 in PA samples. Human PA cell line HP75 was used as a cell model. The biological effects of SNHG6 and miR-944 on HP75 cells were investigated with cell counting kit-8 (CCK-8) assay, Transwell assay, and scratch healing assay *in vitro*, respectively. Markers of EMT, including E-cadherin and vimentin, were detected by Western blot. Interactions between SNHG6 and miR-944, miR-944 and *RAB11A* were determined by bioinformatics analysis, qRT-PCR, and dual luciferase reporter assay.

**Results:**

SNHG6 was significantly upregulated in IPA samples, whereas miR-944 was downregulated. SNHG6 markedly promoted viability, migration, invasion, and EMT of PA cells, whereas miR-944 transfection had the opposite effects. SNHG6 could downregulate miR-944, and there was a negative correlation between SNHG6 expression and miR-944 expression in IPA samples. Besides, it was confirmed that miR-944 could pair with the 3′-untranslated region of *RAB11A* and repress its expression.

**Conclusions:**

This study authenticates that the SNHG6/miR-994/*RAB11A* axis plays a crucial role in regulating proliferation, migration, invasion, and EMT of IPA cells. SNHG6 and miR-994 can serve as novel valuable therapeutic targets for IPA.

## Introduction

Pituitary adenomas (PAs), 10%–15% of all cranial tumors, are the third most common brain tumor.^1^ Although most PAs are benign, their location often leads to compression of adjacent structures.^2^ Surgery remains the first line of treatment for the majority of patients, which cannot effectively control invasive pituitary adenoma (IPA).^3^ Encouraging progress in diagnosis and therapy has been achieved in recent years, and the 5-year overall survival rate of PA patients has been continuously improving.^4^ However, an in-depth understanding of the pathogenesis of IPA will help to determine more effective treatment strategies.

Long noncoding RNA (lncRNA), a class of noncoding RNA with a length of >200 nucleotides, is widely involved in carcinogenesis and cancer progression.^5^ LncRNA is reported to play a crucial role in the pathogenesis of PA. For instance, lncRNA CCAT2, activated by *E2F1*, plays a cancer-promoting role in PA by interacting with PTTG1.^6^ LncRNA small nucleolar RNA host gene 6 (SNHG6) was validated to be oncogenic in several cancers, including hepatocellular carcinoma and colorectal cancer.^7,8^ Unfortunately, the biological function and mechanism of SNHG6 in IPA remain poorly understood.

MicroRNA (miRNA) is a group of endogenous small noncoding RNA molecules that participates in various biological processes through targeted binding to the 3′-untranslated region (3′-UTR) of mRNA.^9^ Many miRNAs have oncogenic or tumor-suppressive properties. For example, miR-21 can predict the poor prognosis of osteosarcoma patients^10^; miR-148a and miR-375 emerge as predictive biomarkers for early diagnosis of laryngeal cancer.^11^ MiR-944 promotes the progression of endometrial cancer^12^; however, it inhibits gastric cancer metastasis through regulating MACC1/Met/AKT signaling pathway.^13^ Nonetheless, the role of miR-944 in PA needs further exploration.

Recently, ongoing research has discovered a novel regulatory mechanism in cancer biology: lncRNA can function as competitive endogenous RNA (ceRNA) to reduce its expression. For example, lncRNA XIST acts as a molecular sponge of miR-194-5p to regulate the expression of MAPK1 in liver cancer cells^14^; in gastric cancer, SNHG6 enhances cell proliferation and epithelial–mesenchymal transition (EMT) by decoying miR-101-3p.^15^ In this study, we demonstrate that SNHG6 was overexpressed in IPA tissues, and it could repress the expression of miR-944 as a ceRNA. We also prove that SNHG6 promoted the EMT of HP75 cells, whereas miR-944 had an opposite effect. In addition, *RAB11A*, an oncogene reported to increase the invasiveness of PA, was a target gene of miR-944. In conclusion, our study clarified the roles of SNHG6 and miR-944 in regulating the invasiveness of PA cells, providing new clues for PA diagnosis and treatment.

## Materials and Methods

### Tissue specimens

Noninvasive pituitary adenoma (NIPA) tissues (*n* = 30) and IPA tissues (*n* = 30) from patients in Quzhou People's Hospital were collected (Supplementary Table S1 and Supplementary Fig. S1). Samples were stored in liquid nitrogen at −196°C for subsequent experiments. Written informed consent from all patients involved was obtained. The collection and use of human tissue samples in this study were approved by the Ethics Review Board of Quzhou People's Hospital (Approval No. 20170024).

### Cell culture

Human PA cell line HP75 was provided by ScienCell Research Laboratories. HP75 cells were cultured in Dulbecco's modified Eagle's medium/Ham's nutrient mixture F12 (DMEM/F12; Gibco, Grand Island, NY) containing 10% fetal bovine serum (FBS; HyClone, Logan, UT), 100 U/mL penicillin and 100 mg/mL streptomycin (Sigma, St. Louis, MO) in 5% CO_2_ at 37°C.

### Quantitative real-time polymerase chain reaction

Total RNA was extracted from cells with TRIzol reagent (Invitrogen, Shanghai, China). In accordance with the manufacturer's instructions, PrimeScript™ RT Reagent kit (Invitrogen, Shanghai, China) was adopted to reversely transcribe RNA into cDNA. Bio-Rad CFX 96 PCR system and SYBR Premix Ex Taq kit (Takara, Dalian, China) were used for quantitative real-time polymerase chain reaction (qRT-PCR). GAPDH was the internal reference for detecting the expression levels of SNHG6 and *RAB11A*, and U6 was the internal reference for detecting miR-944. Statistics analyses were performed using 2^−ΔΔCt^ method. The primer sequences are given in Table 1.

**Table 1.** Quantitative Real-Time Polymerase Chain Reaction Primer Sequences

Name	Primer sequences
SNHG6	Forward: 5′-CTCTGCGAGGTGCAAGAAAG-3′
	Reverse: 5′-AATACATGCCGCGTGATCCT-3′
GAPDH	Forward: 5′-CGCTCTCTGCTCCTCCTGTTC-3′
	Reverse: 5′-ATCCGTTGACTCCGACCTTCAC-3′
miR-944	Forward: 5′-GCGGCGGAAATTATTGTACATC-3′
	Reverse: 5′-ATCCAGTGCAGGGTCCGAGG-3′
U6	Forward: 5′-CTCGCTTCGGCAGCACA-3′
	Reverse: 5′-AACGCTTCACGAATTTGCGT-3′

### Cell transfection

shRNA, overexpression plasmid, microRNA mimics, and miRNA inhibitors were designed and constructed by GenePharma (Shanghai, China). The oligonucleotides were transfected into PA cells at a final concentration of 50 nM using the Lipofectamine^®^ 2000 reagent (Invitrogen, Carlsbad, CA) following the manufacturer's instructions. HP75 cells were washed three times using PBS buffer, trypsinized for 2 min, and inoculated into a 12-well plate with a cell density of 1 × 10^6^ cells/mL. Transfection reagents were diluted with serum-free medium and incubated at 37°C for 20 min. shRNAs and plasmids were also diluted with serum-free medium, incubated at room temperature for 5 min, mixed with the same volume of transfection reagent, and then added into the cells. After 12 h, the transfected cells were observed, and the serum-free medium was replaced with the complete medium. After 48 h of continuous culture, RNA was extracted from the cells, and qRT-PCR was performed to verify the transfection efficiency. In the same way, miR-944 mimics and control miRNA mimics were transfected into PA cell line HP75. The shRNA sequences used for knocking down SNHG6 were as follows: shRNA, 5′-GCATATAGGTTGCTGTAGA-3′. The sh-NC sequence used was 5′-CGATAATGCTGTTAGGAGT-3′.

### Cell counting kit-8 assay

Cell proliferation was detected by cell counting kit-8 (CCK-8) regent (Beyotime, Shanghai, China). HP75 cells were inoculated into 96-well plates (cell density: 2 × 10^3^ cells/well) and cultured for 12 h. Subsequently, HP75 cells were incubated with CCK-8 reagent (10 μL/well) for 1 h at 37°C. Then the absorbance was measured at 450 nm. Subsequently, the cells were measured at intervals of 24 h for consecutive 4 d.

### Transwell assay

Transwell chambers (8 μm pore size; Corning, Beijing, China) were used for the evaluation of migration and invasion of HP75 cells. Matrigel (BD Biosciences, Franklin Lakes, NJ) was used in the invasion assay, but not in the migration assay. HP75 cells were harvested and centrifuged, followed by being resuspended and dispersed. About 5 × 10^4^ cells with serum-free medium were added in the upper chamber; medium containing 10% FBS was added in the lower chamber. After culturing at 37°C for 24 h, cells that failed to migrate or invade were removed from the upper chamber. The membrane was taken and fixed with 4% paraformaldehyde for 10 min before dying with 0.5% crystal violet. Finally, the membrane was rinsed with tap water and the cells were counted under an inverted microscope.

### Dual luciferase reporter gene assay

The fragment of SNHG6 sequence containing the predicted bind sites of miR-944 was inserted into the pmirGlO Dual-luciferase vector (Promega, Madison, WI) to generate the reporter vector with the wild-type SNHG6 (WT-SNHG6). The mutated type SNHG6 (MUT-SNHG6) reporter vector contained the mutated binding sites. HP75 cells were cotransfected with 50 ng of WT-SNHG6 or MUT-SNHG6 and miR-944 mimics or control miRNA mimics using Lipofectamine 2000. After 48 h of transfection, the relative luciferase activity of each group was determined using dual-luciferase reporter gene assay kit (Promega). With the same method, the binding relationship between miR-944 and the 3′UTR of RAB11A was determined.

### Western blot

Cells or tissues were lysed with RIPA lysis (Beyotime, Shanghai, China) to collect total protein. The protein concentration was determined by BCA protein assay kit (Beyotime, Haimen, China). The protein was separated using 10% sodium dodecyl sulfate–polyacrylamide gel electrophoresis and transferred to polyvinylidene fluoride membrane. After being blocked with 5% skim milk, the membrane was incubated with primary antibodies: anti-vimentin (1:1000, # 5741; CST), anti-E-cadherin (1:1000, # 3195; CST), anti-RAB11A (1:500, ab128913; Abcam), and anti-β-actin (1:2000, ab179467; Abcam). Subsequently, the membrane was incubated with the secondary antibody: goat anti-rabbit (1:5000, ab6721; Abcam) or goat anti-mouse (1: 5000, ab6728; Abcam). Band development was carried out utilizing ECL kit (Amersham Pharmacia Biotech, Little Chalfont, United Kingdom) and photographs were taken using ImageQuant LAS 4000 micro biomolecular imager (GE Healthcare).

### Wound-healing assays

Cells were resuspended and inoculated in six-well plates (1 × 10^5^ cells/well) and cultured. After the cell confluency reached ∼90%, cell monolayer was subsequently scratched with a 200 μL sterile pipette tip. The locations of cells at 0 and 24 h in the scratch area were photographed, and the percentage of open space covered by migrated cells was calculated.

### Statistical methods

SPSS software (Version 20.0; Chicago, IL) was used for statistical analysis. All data were expressed as mean ± SD. Student's *t*-test was used for statistical analysis. The difference was statistically significant with *p* < 0.05.

## Results

### SNHG6 was significantly upregulated in IPA tissues, and it was correlated with the expression of vimentin and E-cadherin

**FIG. 1.**
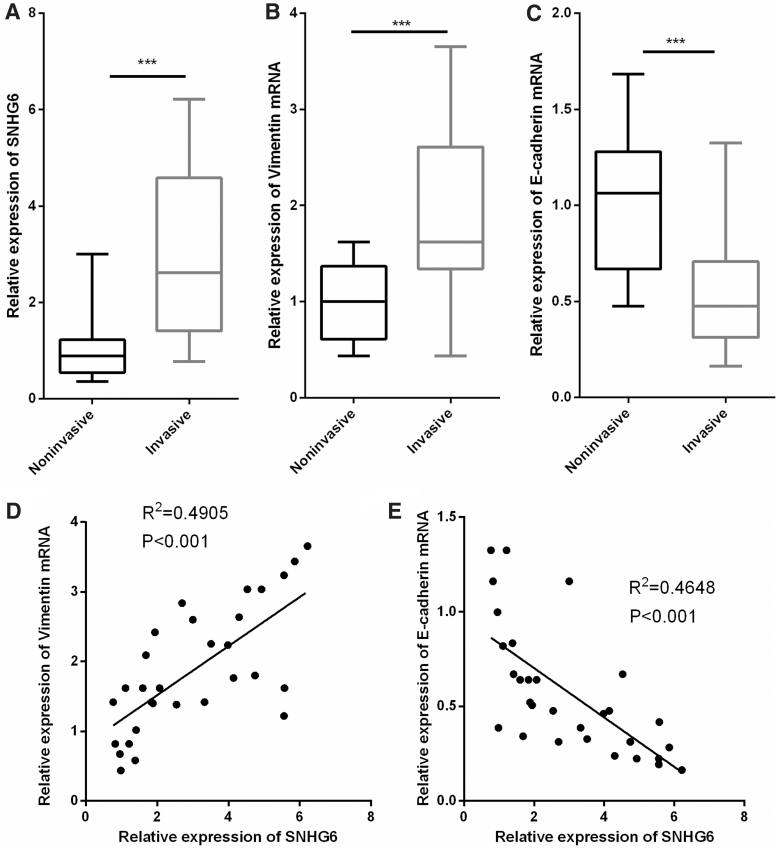
SNHG6 was significantly upregulated in IPA. **(A–C)** qRT-PCR was performed to detect the expression levels of SNHG6, vimentin, and E-cadherin in PA tissues and IPA tissues, respectively. **(D, E)** The correlations between SNHG6 expression and vimentin expression, SNHG6 expression and E-cadherin expression in IPA tissues were analyzed. ***p < 0.001. IPA, invasive pituitary adenoma; qRT-PCR, quantitative real-time polymerase chain reaction.

To determine whether SNHG6 was abnormally expressed in IPA, we performed qRT-PCR to detect the expression level of SNHG6 in IPA tissues and NIPA tissues. As shown, SNHG6 was observed to be significantly upregulated in IPA tissues compared with NIPA tissues (Fig. 1A). We also detected the expression levels of vimentin and E-cadherin, two EMT markers in the samples. As shown, vimentin was markedly upregulated, whereas E-cadherin was notably downregulated in IPA tissues, which was consistent with the biological properties with IPA (Fig. 1B, C). Of importance, we demonstrated a positive correlation between SNHG6 expression and vimentin expression, and a negative association between the expressions of SNHG6 and E-cadherin, which indicated potential regulatory relationships between SNHG6 and vimentin, SNHG6 and E-cadherin (Fig. 1D, E).

### Knockdown of SNHG6 markedly inhibited proliferation, migration, invasion, and EMT of HP75 cells

**FIG. 2.**
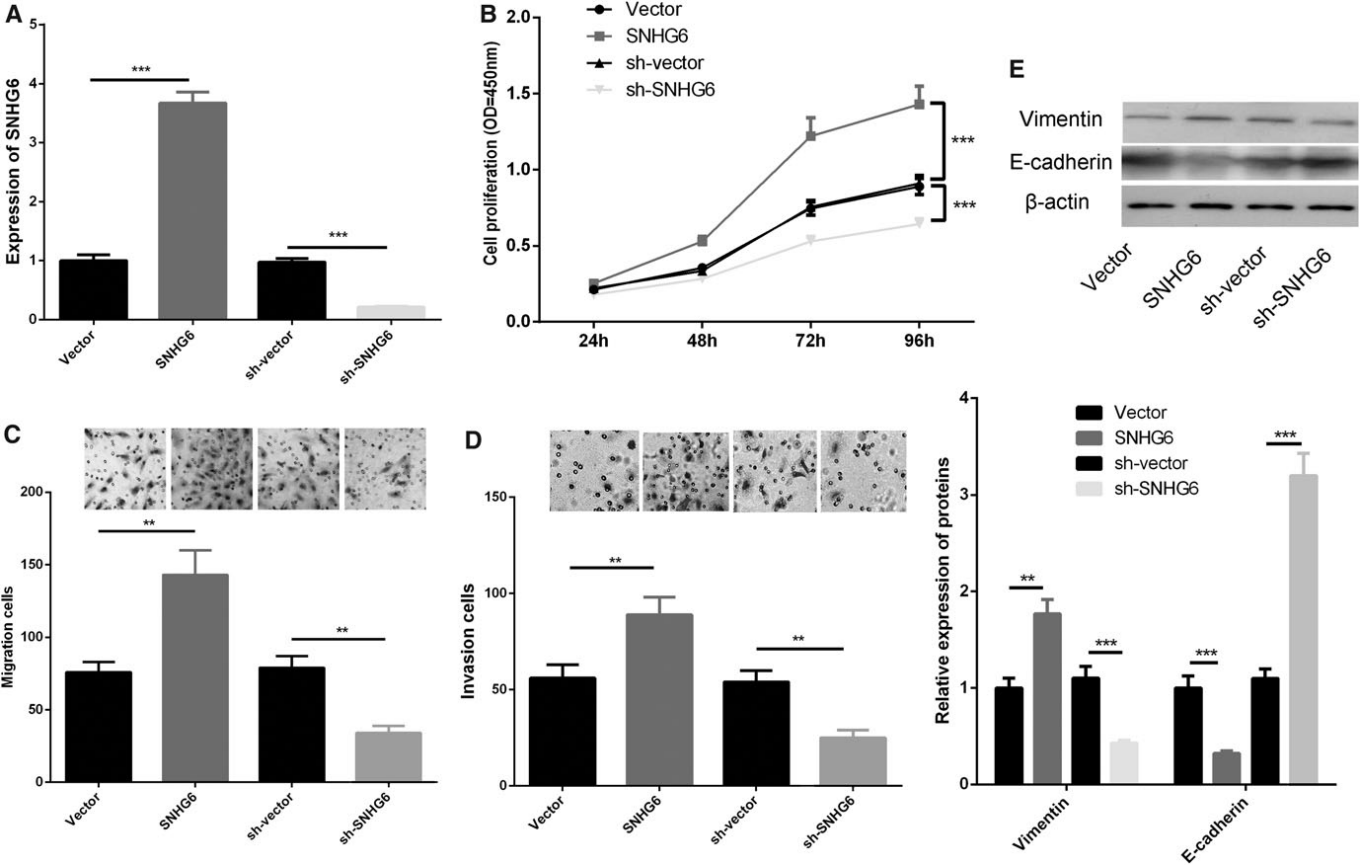
SNHG6 regulated the viability, migration, invasion, and EMT of HP75 cells. **(A)** SNHG6 overexpression model and knockdown model were established, respectively, using HP75 cells, and the expression level of SNHG6 was detected by qRT-PCR. **(B)** CCK-8 assay was conducted to detect the proliferation of HP75 cells. **(C, D)** Transwell migration assay was used to detect migration and invasion of HP75 cells. **(E)** Western blot was carried out to detect the expressions of E-cadherin and vimentin after SNHG6 was upregulated or downregulated in HP75 cells. **p < 0.01; ***p < 0.001. CCK-8, cell counting kit-8; EMT, epithelial–mesenchymal transition.

To study the biological effect of SNHG6 on PA cells, we constructed SNHG6 overexpression and knockdown models with SNHG6 overexpression plasmid and shRNA, respectively, with HP75 cells, and verified the expression of SNHG6 with qRT-PCR (Fig. 2A). CCK-8 assay demonstrated that SNHG6 overexpression could significantly promote the proliferation of HP75 cells, whereas SNHG6 knockdown suppressed the proliferation (Fig. 2B). In addition, Transwell assays and scratch healing assay indicated that SNHG6 overexpression markedly facilitated HP75 cell migration and invasion, but its knockdown showed opposite effects (Fig. 2C, D and Supplementary Fig. S2). Of importance, Western blot showed that SNHG6 overexpression resulted in increased expression of vimentin protein and decreased expression of E-cadherin protein, whereas its knockdown exerted opposite effects (Fig. 2E). The above results validated that SNHG6 was a crucial regulator in the proliferation, migration, invasion, and EMT of PA cells.

### SNHG6 regulated the expression of miR-944

**FIG. 3.**
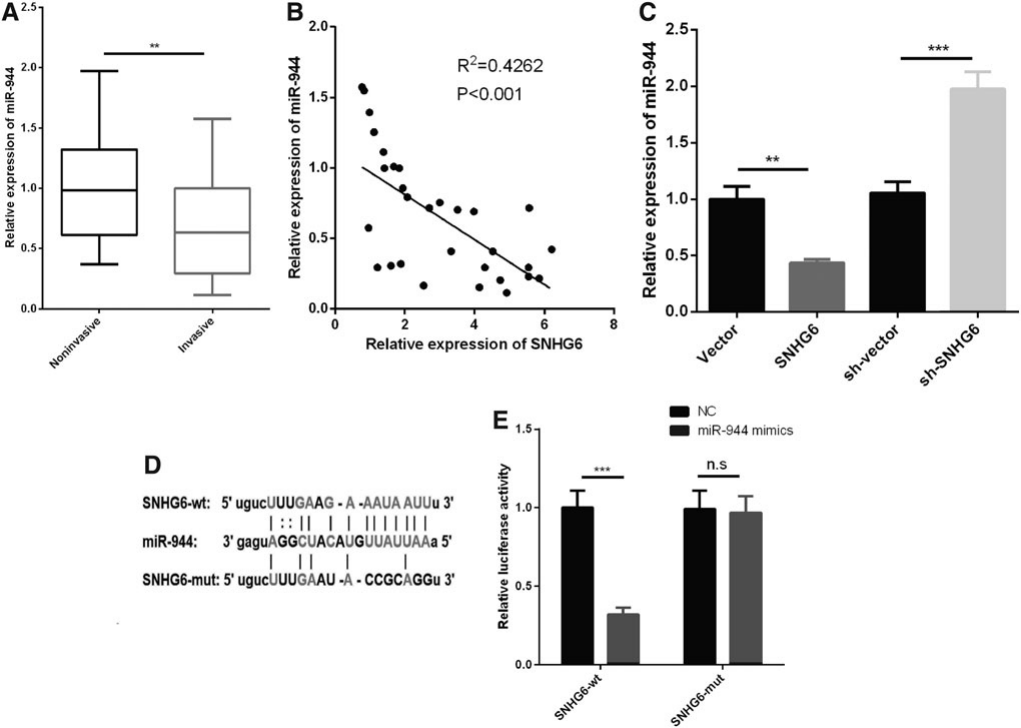
miR-944 was downregulated in IPA tissues and negatively regulated by SNHG6. **(A)** The expression of miR-944 in IPA tissues and NIPA tissues was detected by qRT-PCR. **(B)** The correlation between SNHG6 expression and miR-944 expression in IPA tissues was analyzed. **(C)** qRT-PCR was performed to detect the expression of miR-944 after SNHG6 was upregulated or downregulated in HP75 cells. **(D)** Schematic diagram of binding sites between wild-type or mutant-type SNHG6 and miR-944. **(E)** Dual luciferase reporter assay was used to validate the binding relationship between SNHG6 and miR-944. **p < 0.01; ***p < 0.001; *p* > 0.05. NIPA, noninvasive pituitary adenoma; n.s, no significance.

Next, we investigated the expression of miR-944 in IPA. qRT-PCR data indicated that miR-944 was significantly downregulated in IPA tissues compared with NIPA tissues (Fig. 3A). We also demonstrated that miR-944 expression was negatively correlated with SNHG6 expression in IPA tissues (Fig. 3B), and SNHG6 could negatively regulate the expression level of miR-944 in HP75 cells (Fig. 3C). Of interest, by searching Starbase database, we noticed that SNHG6 contains a conserved target site of miR-944 (Fig. 3D). Dual luciferase reporter assay indicated that transfection of miR-944 observably reduced the luciferase activity of WT-SNHG6, whereas no significant effect could be found on the luciferase activity of MUT-SNHG6 (Fig. 3E). From the above data, we confirmed the regulatory relationship between SNHG6 and miR-944, which suggested that SNHG6 was a ceRNA for miR-944.

### miR-944 transfection inhibited viability, migration, invasion, and EMT of PA cells

**FIG. 4.**
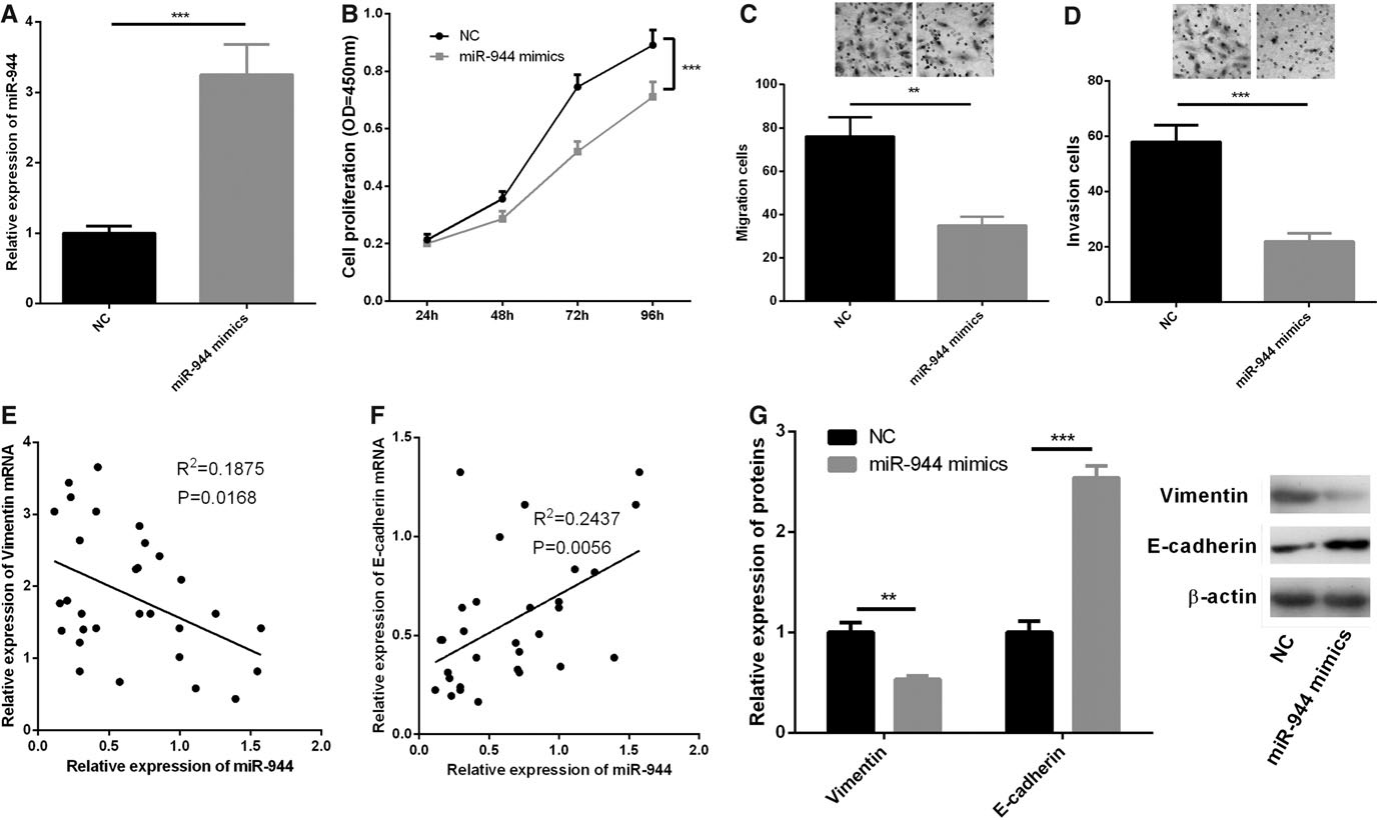
miR-944 transfection inhibited viability, migration, invasion, and EMT of HP75 cells. **(A)** HP75 cells were transfected with control miRNA mimics or miR-944 mimics, and the expression of miR-944 in HP75 cells was detected by qRT-PCR. **(B)** CCK-8 assay was used to detect the proliferation of HP75 cells. **(C, D)** Transwell assay was used to detect the migration and invasion of HP75 cells. **(E, F)** The correlations between miR-944 expression and vimentin expression, miR-944 expression and E-cadherin expression in IPA tissues were analyzed. **(G)** Western blot was carried out to detect the expressions of E-cadherin and vimentin after transfection of miR-944 mimics in HP75 cells. **p < 0.01; ***p < 0.001.

To determine the effects of miR-944 on proliferation, migration, invasion, and EMT of PA cells, we transfected miR-944 mimics into HP75 cells and successfully constructed an miR-944 overexpression model (Fig. 4A). CCK-8 assay showed that overexpression of miR-944 could significantly restrain the proliferation of HP75 cells (Fig. 4B). In Transwell migration and invasion experiments, we observed that overexpressed miR-944 markedly repressed HP75 cell migration and invasion (Fig. 4C, D). We also demonstrated a negative correlation between miR-944 expression and vimentin expression and a positive correlation between miR-944 expression and E-cadherin expression (Fig. 4E, F). In addition, Western blot indicated that overexpression of miR-944 resulted in decreased protein expression of vimentin and increased protein expression level of E-cadherin in HP75 cells (Fig. 4G). The above results showed that miR-944 had inhibitory effects on the growth, migration, invasion, and EMT of PA cells.

### SNHG6/miR-944 axis regulated the biological behaviors of PA cells

**FIG. 5.**
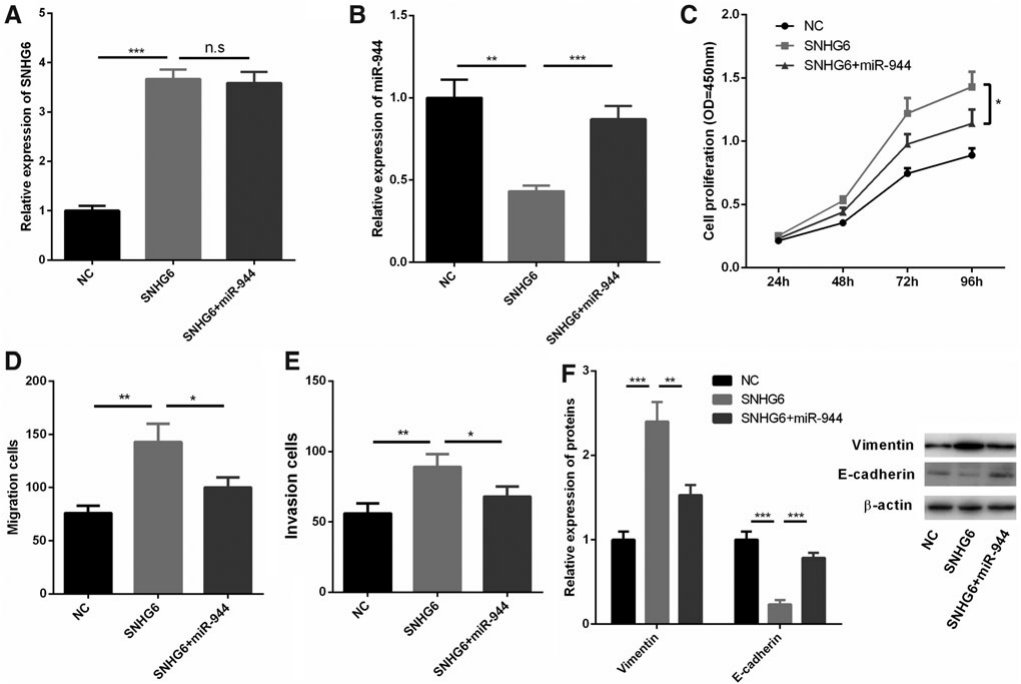
SNHG6/miR-944 axis regulated the biological behaviors of HP75 cells. **(A, B)** HP75 cells were transfected with control plasmids, SNHG6 overexpression plasmids, or SNHG6 overexpression plasmids+miR-944 mimics, respectively, and the expression levels of SNHG6 and miR-944 were detected by qRT-PCR. **(C)** CCK-8 assay was used to detect the proliferation of HP75 cells. **(D, E)** Transwell migration assay was used to detect migration and invasion of HP75 cells. **(F)** Western blot was carried out to detect the expressions of E-cadherin and vimentin in HP75 cells of different groups. *p < 0.05; **p < 0.01; ***p < 0.001; ^n.s^*p* > 0.05.

To verify the effect of SNHG6/miR-944 axis on PA proliferation and metastasis, we further transfected miR-944 mimics into HP75 cells with SNHG6 overexpression. It was found that miR-944 was downregulated in HP75 cells transfected with SNHG6, but this effect was reversed by cotransfection with miR-944 mimics (Fig. 5A, B). CCK-8 assay signified that miR-944 could partially offset the promotion of proliferation, migration, and invasion of HP75 cells induced by SNHG6 (Fig. 5C–E). Western blot also showed that the changes of vimentin expression and E-cadherin expression induced by SNHG6 were reversed by the cotransfection of miR-944 (Fig. 5F). Collectively, these data further confirmed the role of SNHG6/miR-944 axis in regulating the malignant phenotypes of PA.

### MiR-944 directly targeted *RAB11A* in PA cells

**FIG. 6.**
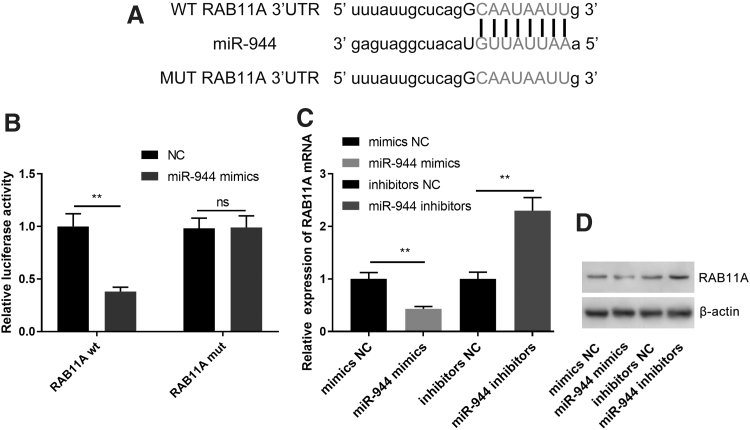
*RAB11A* was a direct target of miR-944 in PA cells. **(A)** Bioinformatic analysis with miRDB database, starBase and TargetscanHuman database (TargetScanHuman 7.2) predicted that the 3′UTR of *RAB11A* possessed a sequence that was complementary to miR-944. **(B)** MiR-944 overexpression significantly suppressed the luciferase activity of WT-RAB11A 3′-UTR reporter, rather than that of MUT-RAB11A 3′-UTR reporter. **(C, D)** MiR-944 overexpression reduced the expression of RAB11A mRNA and protein in PA cells and miR-944 inhibitors increased the expression level of RAB11A mRNA and protein. **p < 0.01.

To further explore the downstream mechanism through which miR-944 exerted its effects on PA cells, we found that there was a potential binding site between the 3′UTR of *RAB11A* and miR-944 through the analysis of miRDB database, starBase, and TargetscanHuman database (TargetScanHuman 7.2) (Fig. 6A). Dual luciferase reporter assays suggested that miR-944 mimics significantly reduced the luciferase activity of WT-RAB11A 3′-UTR, whereas miR-944 mimics did not change the luciferase activity of MUT-RAB11A 3′UTR (Fig. 6B). Moreover, qRT-PCR and Western blot also indicated that miR-944 mimics markedly inhibited RAB11A at both mRNA and protein levels in HP75 cells, whereas downregulation of miR-944 increased RAB11A expression (Fig. 6C, D). Collectively, these findings demonstrated that *RAB11A* was a downstream target of miR-944 in PA.

## Discussion

Diverse lncRNAs show abnormal expressions in tumors, many of which affect the malignant phenotypes of tumors, including cell proliferation, apoptosis, and metastasis.^16,17^ In detail, lncRNA VELUCT has a strong regulatory effect on the viability of lung cancer cells^18^; lncRNA MEG3 inhibits the proliferation and metastasis of gastric cancer through regulating p53 signaling.^19^ LncRNA CCAT1 promotes the progression of glioma by regulating miR-181b,^20^ and LINC00460 targets miR-539/MMP-9 axis to facilitate the progression of meningiomas.^21^ In this study, we explored the expression characteristics and functions of SNHG6 in PA. We observed that the expression of SNHG6 in IPA tissues was significantly higher than that in NIPA tissues. Furthermore, the proliferation, migration, and invasion of PA cells could be notably restrained by the knockdown of SNHG6 but promoted by overexpressed SNHG6. These data suggested that SNHG6 could be an important regulator of invasiveness in PA.

EMT is predictive of the tumor invasion and metastasis. During this process, tumor cells acquire stronger ability to migrate, invade, and proliferate.^22^ LncRNA is also involved in the regulation of EMT. For example, knockdown of lncRNA TUG1 can promote the expression of miR-384 to inhibit EMT and thus suppress the progression of nasopharyngeal carcinoma^23^; downregulation of lncRNA-ATB can repress EMT of breast cancer cells by increasing the expression of miR-141-3p.^24^ E-cadherin and vimentin are reported as EMT markers.^25^ In this study, we demonstrated that SNHG6 could increase the invasiveness of PA cells by inducing EMT.

MiR-944 functions as a tumor suppressor in multiple cancers. In colorectal cancer, it suppresses cancer progression and is likely to be used as a prognostic predictor^26^; it also inhibits the metastasis of breast cancer cells by targeting SIAH1 and PTP4A1.^27^ Consistent with previous reports, our data suggested that miR-944 was significantly downregulated in IPA tissues. In addition, we also proved that miR-944 overexpression significantly inhibited proliferation, migration, invasion, and EMT of PA cells. The above results confirmed the antitumor effect of miR-944 in PA cells.

LncRNA, as ceRNA, can regulate the malignant biological behaviors of cancer cells including EMT, through sponging miRNA. For example, lncRNA XIST is involved in transforming growth factor-β1-induced EMT of nonsmall cell lung cancer cells by regulating miR-137^28^; lncRNA CCAT1 promoted EMT of ovarian cancer cells by sponging miR-490-3p.^29^ To get insights into the molecular mechanism of SNHG6 to regulate PA progression, we found through bioinformatics analysis that miR-944 was one of the targets of SNHG6, and there was a negative correlation between miR-944 and SNHG6 expressions in PA tissue. Moreover, overexpression of SNHG6 reduced the expression of miR-944 in PA cells. These results validated the regulatory relationship between SNHG6 and miR-944, partly explaining the mechanism by which SNHG6 facilitated the progression of PA.

RAB11A belongs to the Rab family of the small GTPase superfamily, which plays a role in the process of protein transport, and is involved in many biological processes including cell proliferation, migration, and invasion.^30^ RAB11A is overexpressed in thyroid cancer, nonsmall cell lung cancer, and invasive pituitary tumors.^31–33^ In terms of mechanism, RAB11A is crucial for the activation of Akt signaling.^34^ In addition, RAB11A plays an oncogenic role in PA by activating the Wnt/β-catenin signaling pathway.^31^ In addition, RAB11A is reported to be modulated by multiple miRNA, such as miR-21-5p and miR-452.^35,36^ In this study, RAB11A was identified as a target gene of miR-944, suggesting that it was a crucial downstream effector of SNHG6/miR-944 axis in IPA

To sum up, the upregulation of SNHG6 is a characteristic of IPA, and it can facilitate the proliferation, migration, invasion, and EMT of PA cells. In addition, miR-944 plays a tumor-suppressive role in PA, and SNHG6 can negatively regulate it. What is more, SNHG6/miR-944 axis may improve the malignancy of PA cells by regulating RAB11A. This study helps clarify the pathogenesis of IPA and provides new theoretical basis for diagnosis and treatment of PA.

## Ethics Statement

Our study was approved by the ethics review board of Quzhou People's Hospital.

## Data Availability Statement

The data used to support the findings of this study are available from the corresponding author upon request.

## Supplementary Material

Supplemental data

Supplemental data

Supplemental data
