# Supplementary material for: LncRNA SNHG6 Induces Epithelial–Mesenchymal Transition of Pituitary Adenoma Via Suppressing MiR-944
Source: Cancer Biother Radiopharm. 2022 May 10;37(4):246–55. doi: 10.1089/cbr.2020.3587 (PMC9127839; doi:10.1089/cbr.2020.3587)
Supplement: Supplemental data [file Supp_TableS1.docx]

|  | Invasive pituitary adenoma (n = 30) | Non-invasive pituitary adenoma (n = 30) |
| --- | --- | --- |
| Mean age (years) | 44.5 | 41.8 |
| Gender (man/women) | 13/17 | 12/18 |
| GH-omas | 14 | 16 |
| ACTH-omas | 5 | 7 |
| NFPAs | 9 | 6 |
| PRL-omas | 2 | 1 |

**Supplementary Table 1. The clinical information of the patients in the study.**
