# Supplementary figures and images for: LncRNA SNHG6 Induces Epithelial–Mesenchymal Transition of Pituitary Adenoma Via Suppressing MiR-944
Source: Cancer Biother Radiopharm. 2022 May 10;37(4):246–55. doi: 10.1089/cbr.2020.3587 (PMC9127839; doi:10.1089/cbr.2020.3587)

Supplementary Figure 1. Representative MRI iamages of NIPA adenoma (left) and IPA (right).


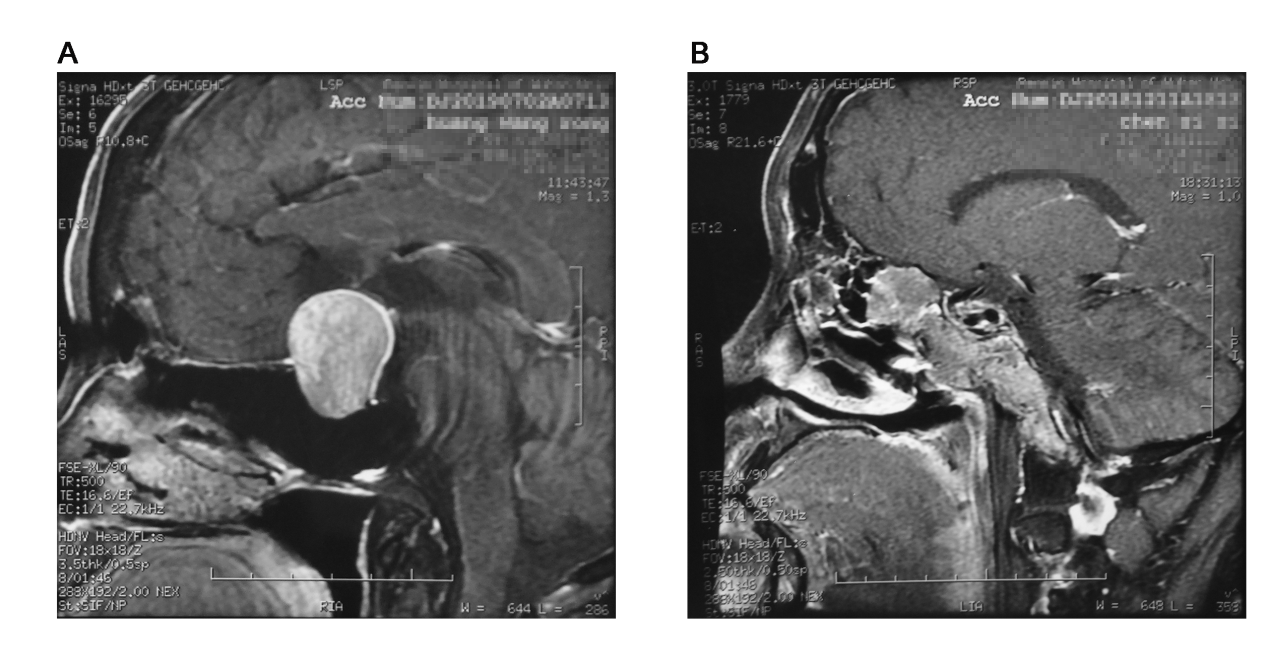

Supplement: Supplemental data [file Supp_FigS1.docx]

Supplementary Figure 2. The cell mobility was detected by scratch healing assay.


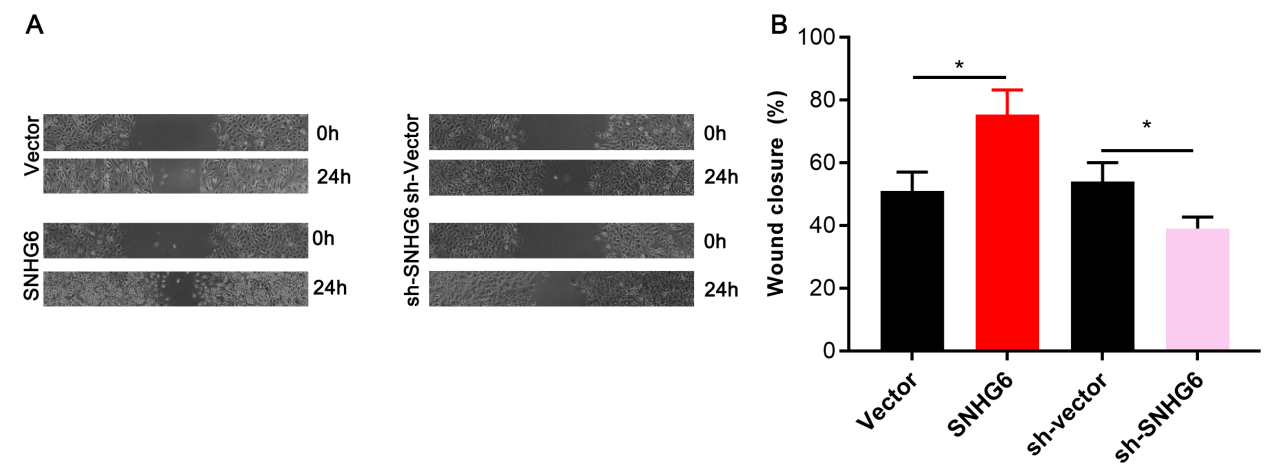

Supplement: Supplemental data [file Supp_FigS2.docx]
